# Multiple Glacial Refugia of the Low-Dispersal Ground Beetle *Carabus irregularis*: Molecular Data Support Predictions of Species Distribution Models

**DOI:** 10.1371/journal.pone.0061185

**Published:** 2013-04-04

**Authors:** Katharina Homburg, Claudia Drees, Martin M. Gossner, László Rakosy, Al Vrezec, Thorsten Assmann

**Affiliations:** 1 Leuphana University Lüneburg, Institute of Ecology, Lüneburg, Germany; 2 University of Hamburg, Biocentre Grindel and Zoological Museum, Behavioural Biology, Hamburg, Germany; 3 Technische Universität München, Department of Ecology and Ecosystem Management, Freising-Weihenstephan, Germany; 4 Babeş-Bolyai University Cluj-Napoca, Faculty of Biology and Geology, Cluj-Napoca, Romania; 5 National Institute of Biology, Department of Freshwater and Terrestrial Ecosystems Research, Ljubljana, Slovenia; Centre National de la Recherche Scientifique, France

## Abstract

Classical glacial refugia such as the southern European peninsulas were important for species survival during glacial periods and acted as sources of post-glacial colonisation processes. Only recently, some studies have provided evidence for glacial refugia north of the southern European peninsulas. In the present study, we combined species distribution models (SDMs) with phylogeographic analyses (using mitochondrial DNA = mtDNA) to investigate if the cold-adapted, stenotopic and flightless ground beetle species, *Carabus irregularis*, survived the Last Glacial Maximum (LGM) in classical and/or other refugia. SDMs (for both a western European and for a Carpathian subgroup) were calculated with MAXENT on the basis of 645 species records to predict current and past distribution patterns. Two mtDNA loci (CO1 and ND5, concatenated sequence length: 1785 bp) were analyzed from 91 *C. irregularis* specimens to reconstruct the phylogeography of Central and eastern European populations and to estimate divergence times of the given lineages. Strong intra-specific genetic differentiation (inter-clade Φ_ST_ values ranged from 0.92 to 0.99) implied long-term isolation of major clades and subsclades. The high divergence between the nominate subspecies and the Carpathian subspecies *C. i. montandoni* points to two independent species rather than subspecies (K-2P distance 0.042 ± 0.004; supposed divergence of the maternal lineages dated back 1.6 to 2.5 million years BP) differing not only morphologically but also genetically and ecologically from each other. The SDMs also inferred classical as well as other refugia for *C. irregularis*, especially north of the Alps, in southeastern Europe and in the Carpathians. The coincidences between the results of both methods confirm the assumption of multiple glacial refugia for the studied species and the usefulness of combining methodological approaches for the understanding of the history of low-dispersal insect species.

## Introduction

The glaciers and permafrost grounds of the Quaternary ice ages shaped distribution patterns in biodiversity, as they caused species extinctions as well as the retraction of species' distribution ranges. Glacial refugia were important for species survival in glacial and interglacial periods and hosted many species which had recently been widespread across Europe [1]. Temperate and warm-adapted species in particular are assumed to have colonised central and northern parts of Europe after the Last Glacial Maximum (LGM), originating from classical refugia such as the southern European peninsulas (Iberian, Apennine and Balkan) [1], [2]. Cold-adapted species that had recently inhabited alpine and arctic habitats are assumed to have survived in the margins of southern European mountain chains [3]. In recent decades, palaeontological, palynological and phylogeographic studies have provided evidence for glacial refugia far north of the southern European peninsulas and the Alps (so-called cryptic refugia) [4], [5]. In contrast to the species which were widespread across Europe [6], [7], species with restricted and disjunct ranges in the European low and high mountain chains are poorly studied with regard to spatial patterns in genetic variation. However, some mountain plant species inhabiting the Alps and geographically relatively close mountain systems (e.g. the Central European mountain ranges, the Carpathians) show high genetic differentiation [8], [9]. Zoological studies have covered arthropod species with high dispersal abilities (e.g. due to flight activity or ballooning): Butterflies [10] and spiders [11] show low level differentiations and indicate young and even post-glacial splits between populations from the Alps and adjoining high and low mountain ranges [12]. In contrast, low-dispersal species are likely to show stronger genetic differentiation patterns due to the absence of gene flow (e.g. flightless carabids; [13], [14]); however, the population histories of these species have been studied only insufficiently to date.

New study methods such as species distribution models (SDMs) have become useful tools to predict potential habitat distribution of species under current climate conditions and – when analysed in conjunction with palaeoclimatic data – to project species' past distributions [15]. Many studies detected classical as well as cryptic refugia using past predictive models (e.g. [16], [17]). Unaffected by incomplete sampling, the extinction of genetic variants and large-scale range shifts of species, past predictive models can contribute to the localization and sizing of species' glacial refugia [18]. Consequently, past predictive models and phylogeographic analyses can be used complementarily to develop reasonable scenarios of species' range retractions during glaciations and of range expansions during interglacial stages.

In this study we applied a combined approach using modelling and of phylogeographic analysis of two mitochondrial DNA loci (CO1 and ND5) to investigate the history of the flightless ground beetle species, which occurs in a disjunct range across high- and low-altitude mountains of Central and eastern Europe: *Carabus irregularis.* We aimed to test (i) whether the cold-adapted species survived the LGM in classical and/or cryptic refugia and (ii) whether the low-dispersal species shows old phylogenetic groups and high intra-specific variation. Since the CO1 locus has been successfully used in barcoding [19], we also checked (iii) whether the phylogeny of *Carabus irregularis* is consistent with the intra-specific taxonomy of the species.

We addressed the following specific study questions: (1) Where did *C. irregularis* find suitable climate conditions (refugia) to survive the LGM? (2) Does the species' genetic differentiation pattern provide molecular evidence for low dispersal and for the detected potential glacial refugia? (3) Is the phylogeny of *C. irregularis* congruent with its taxonomic subspecies delineation?

## Materials and Methods

### Study species

**Figure 1:**
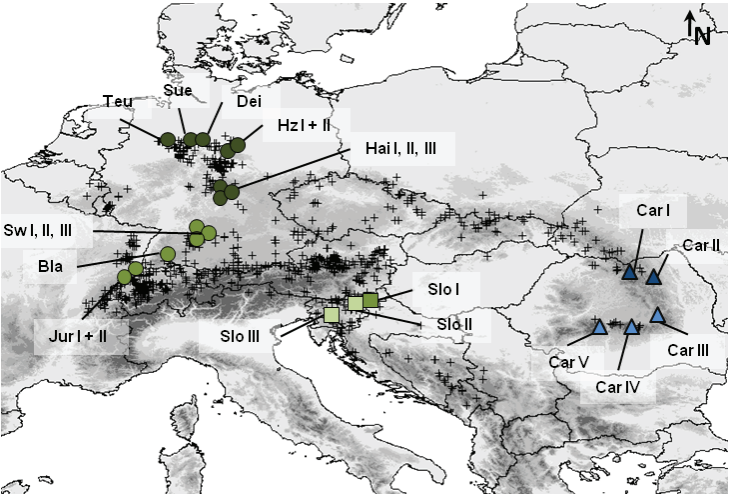
Species records of *Carabus irregularis* used for species distribution modelling and populations sampled for genetic analyses. SDMs included species records displayed as small crosses. Genetic analyses included populations of the three subspecies – *C. i. irregularis* (circles), *C. i. bucephalus* (squares) and *C. i. montandoni* (triangles). Genetic analyses resulted in two major clades: a Carpathian clade (Clade A = blue colours) and a European clade (Clade B = green colours). The clades split into five subclades: an eastern Carpathian (Ae = dark blue), a southern Carpathian (As = light blue), a northern European (Bn = dark green), a southern European (Bs = olive green) and a purely Slovenian clade (Bsl = light green).

*Carabus (Platycarabus) irregularis* Fabricius, 1792 occurs in cold and mesic habitats in montane to subalpine altitudes (e.g. beech forests and high mountain meadows). Suitable habitats are predominantly on limestone and are rich in snails, the preferred food of *C. irregularis*. The disjunct distribution of *C. irregularis* ranges from the Teutoburg Forest in the north to the Dinaric Mountains in the south and from the Carpathians in the east to the Jura Mountains in the west (Fig. 1) [20]. In the taxonomic literature, three subspecies are well-accepted even though different names are sometimes used [21]. In this study we follow Casale and Kryzhanovskij [22] and use the following binomials: *C. i. irregularis* s.str. in Central Europe including the northern Alps, *C. i. bucephalus* Kraatz, 1879 in the southeastern Alps and the northern Balkan Peninsula, and *C. i. montandoni* Buysson, 1882 in the Carpathians (Fig. 1). While the distribution ranges of *C. i. irregularis* and *C. i. bucephalus* pass into each other in the southeastern Alps, *C. montandoni* is geographically separated from the other two subspecies (see subspecies affiliation in Table S1). The species is declining in some regions [23], [24] and already extinct in some parts of its former distribution range (e.g. in Belgium [25]).

### Species distribution modelling

In order to predict the potential habitat distribution of the study species under current and past climate conditions, we compiled 1,005 data points of observed occurrences of *C. irregularis* from the literature and databases (e.g. [26]–[33]), from museum and private entomological collections as well as our own field surveys (see complete list of records in Table S1). Since spatial clumping of species records due to different sampling intensities may bias SDMs, redundant localities were excluded by the model software, and the final locality input included 645 records. In addition to the geographic separation, there are also differences in the altitudinal distribution pattern of the two western European subspecies (*C. i. irregularis* and *C. i. bucephalus*) and the Carpathian subspecies (*C. i. montandoni*). According to the compiled species records, *C. i. irregularis* and *C. i. bucephalus* occur in high as well as low altitudes in the western part of its distribution range and on average in lower altitudes (altitudes as low as 250 m a.s.l.; mean altitude of western records = 774 m a.s.l.; standard error (SE) = 13.71; confidence interval (CI) = 26.87; see Fig. S1). From the eastern part of the range (the Carpathian mountain system), however, the species was scarcely recorded from altitudes below 600 m a.s.l. (mean altitude of Carpathian records = 912 m a.s.l.; SE = 45.50; CI = 89.19; see Fig. S1). Due to geographically separated distribution ranges and non-overlapping confidence intervals of the altitudinal distribution of the subspecies, we could not exclude different habitat preferences of the Carpathian subspecies. Thus, species records were split into two sets before modelling: one included occurrences of the Carpathian subspecies, the other comprised occurrences of the two other subspecies from the western part of the distribution range (Central Europe and Balkan). Climate data (monthly temperature and rainfall values) was generated according to Hijmans et al. [34] to 19 bioclimatic variables (BIO1-BIO19; Table S2) which representing factors of climate which are particularly significant in determining species' distributions [18]. For carabids, temperature and humidity variables have been demonstrated to be the most important environmental factors influencing habitat selection [35].

Current climate data (i.e. period 1950–2000) was downloaded with a spatial resolution of 30 arc s (∼1×1 km) from the WorldClim database (http://www.worldclim.org/). Past (Last Glacial Maximum = LGM; 21,000 BP) climate data downscaled from the MIROC general circulation models to the required spatial resolution (2.5 arc min) was provided by the Paleoclimate Modelling Intercomparison Project Phase II (http://pmip2.lsce.ipsl.fr/). Since our SDMs were to include only predictors with high explanatory power and ecological relevance [36], [37], we included only the variables which are most likely to influence the occurrence of *C. irregularis*. Therefore, we extracted all 19 bioclimatic variables at coordinates of species records and performed a principal component analysis (PCA). To reduce multicollinearity, we compiled a correlation matrix and excluded all strongly correlated variables (Pearson's r^2^>0.75; [38]). Final models included five uncorrelated predictor variables: ‘Isothermality’ (BIO3), ‘temperature seasonality’ (BIO4), ‘minimum temperature of coldest month’ (BIO6), ‘precipitation of wettest month’ (BIO13) and ‘precipitation of driest month’ (BIO14) – climatic measures reflecting habitat conditions which are likely to affect the occurrence of *C. irregularis* [30], [31]. The bioclimatic layers were cut to cover the recently documented distribution range of the species sufficiently but not too generous, since large layer extents could have a negative effect on SDM results [39]. The layers used ranged from 30 to 60 decimal degrees North and from 0 to 30 decimal degrees East.

We used the maximum entropy approach [40], which repeatedly performed better than other methods using species presence data in comparative studies [41], [42]. Three sets of SDMs – one with the western record set (hereafter western distribution model), one with only Carpathian records (hereafter Carpathian distribution model) and one model including all records together (hereafter entire distribution model) were generated with MAXENT 3.3.3 (http://www.cs.princeton.edu/~schapire/maxent), with automatically sampled random background points and species records split into 75% model training and 25% model evaluation data. Model quality was evaluated by a bootstrap validation and the Area Under Curve method (AUC>0.7; [43]). The logistic output of 100 model replicates was transformed to species maps showing the potential distribution of *C. irregularis* under current climate conditions and under LGM conditions. Species maps were processed in DIVA-GIS v7.1.7 (http://www.diva-gis.org). We compared the Maxent outputs of the three model sets using the software ENMtools [44]. Niche overlap was quantified by two metrics of niche similarity introduced by Warren et al. (2008) [45]: *D* (Schoener's statistic for niche overlap; [46]) and *I* (derived from Hellinger distance). Both measures range from 0 (no niche overlap) to 1 (identical niches). In addition, a niche identity test was run to evaluate niche similarity between the western and the Carpathian distribution model [44].

### Sample collection and molecular methods

In total 91 specimens of the study species were collected from 23 localities across its entire distribution range (Table 1, Fig. 1). In spite of conscientious efforts and communications with numerous coleopterologists, no fresh beetles were available from certain parts of the distribution range (possibly due to the recent decline mentioned above). Adult beetles were collected from hibernation in autumn or caught by pitfall trapping during the activity periods. The specimens were preserved at –80°C or in pure ethanol at –20°C until DNA extraction.

**Table 1:** Populations of *Carabus irregularis* sampled for genetic analyses and occurrence data used for species distribution models.

Code	Population	Altitude (m a.s.l.)	Geographical coordinates	(Sub-) species	Sample size	Year
Teu	Teutoburg Forest: Wehdeberg (D)	261	52°08.5′N, 08°11.0′E	*irregularis*	5	2010
Sue	Weser Hills: Süntel (D)	231	52°12.2′N, 09°16.3′E	*irregularis*	6	2009
Dei	Weser Hills: Deister (D)	227	52°13.2′N, 09°32.0′E	*irregularis*	1	2010
Hz I	Harz: Schreckenberg (D)	227	51°58.6′N, 10°13.4′E	*irregularis*	6	2010
Hz II	Harz: Fürstenhagen (D)	334	51°49.3′N, 10°09.9′E	*irregularis*	5	2010
Hai I	Hainich: Küllstedt (D)	456	51°16.1′N, 10°14.4′E	*irregularis*	6	2010
Hai II	Hainich: Dingelstädt (D)	455	51°20.2′N, 10°21.9′E	*irregularis*	5	2010
Hai III	Hainich: Mühlhausen (D)	379	51°12.6′N, 10°22.2′E	*irregularis*	4	2011
Sw I	Swabian Mountains: Gomadingen (D)	790	48°23.8′N, 09°27.2′E	*irregularis*	5	2011
Sw II	Swabian Mountains: Münsingen (D)	791	48°23.4′N, 09°30.1′E	*irregularis*	5	2011
Sw III	Swabian Mountains: Engstingen (D)	763	48°22.5′N, 09°20.3′E	*irregularis*	4	2011
Bla	Black Forest: Wutachschlucht (D)	696	47°50.6′N, 08°20.2′E	*irregularis*	6	2010
Jur I	Jura: NO Jougne (F)	1190	46°47.5′N, 06°25.9′E	*irregularis*	1	2010
Jur II	Jura: NW Jougne (F)	1100	46°47.0′N, 06°22.7′E	*irregularis*	5	2010
Slo I	Poljčane: Boč, Formila (SLO)	633	46°17.3′N, 15°37.3′E	*bucephalus*	4	2011
Slo II	Konjiška gora: Stolpnik (SLO)	790	46°20.0′N, 15°22.9′E	*bucephalus*	5	2011
Slo III	S Ljubljana: Krim (SLO)	950	45°54.6′N, 14°27.6′E	*bucephalus*	4	2011
Car I	Carpathians: Rodnei (RO)	1560	47°36.5′N, 24°38.8′E	*montandoni*	2	2009
Car II	Carpathians: Rarău (RO)	1529	47°27.0′N, 25°33.8′E	*montandoni*	5	2009
Car III	Carpathians: Bucegi (RO)	1128	45°29.9′N, 25°30.7′E	*montandoni*	4	2009
Car IV	Carpathians: Făgăraş (RO)	1327	45°38.0′N, 24°36.5′E	*montandoni*	1	2009
Car V	Carpathians: Parang (RO)	1950	45°22.1′N, 23°35.9′E	*montandoni*	2	2009
*creutzeri* 1	Julian Alps, Mangart (SLO)	1905	46°26.1′N, 13°38.5′E	*C. creutzeri*	1	2011
*creutzeri* 2+3	Triglav, Pokljuka (SLO)	1316	46°19.9′N, 13°54.5′E	*C. creutzeri*	2	2011
*depressus* 1+2	Adamello, Val di Daone (I)	2028	45°05.1′N, 10°34.1′E	*C. depressus*	2	2011
*depressus* 3	Valais Alps, Gran San Bernhardo (I)	2223	45°51.8′N, 07°09.5′E	*C. depressus*	1	2011

D: Germany, F: France, RO: Romania, SLO: Slovenia, I: Italy; m a.s.l.: metres above sea level.

Mitochondrial genomes within the genus *Carabus* can be influenced by horizontal gene flow (secondary contact after speciation) [47]–[49]. In natural habitats, *C. irregularis* seems to produce hybrids with related species (members of the same subgenus *Platycarabus*: *C. creutzeri* and *C. depressus*) [50]. Therefore, we used six individuals of the latter species from three different sites as outgroups for rooting phylogenetic trees and for checking the reliability of mitochondrial DNA (mtDNA) as a phylogenetic marker. In comparative studies, the two different mitochondrial genes studied, CO1 and ND5, have outperformed nuclear markers in terms of giving a reliable estimates in timing of splits and phylogenetic reconstruction because of their maternal heritability [51]. Moreover, CO1 is a valuable barcoding marker in animals [19] which makes it possible to study the performance of such a marker system on an infraspecific level.

Genomic DNA was extracted from one femur of each specimen by CTAB (Cetyldimethylethyl-Ammoniumbromid) lysis [52], where we reached a DNA concentration of min. 200 ng/ µl. Two regions of mtDNA, Cytochrome c Oxidase I (CO1) and NADH Dehydrogenase 5 (ND5), were amplified and sequenced using the primers Jerry (C1-J-2183) 5′-CAA CAT TTA TTT TGA TTT TTT G-3′ and Pat (L2-N-3014) 5′-TCC AAT GCA CTA ATC TGC CAT ATT A-3′ [53] for CO1 and ND5-His (V1.06-1) 5′-CCT GTT TCT GCT TTA GTT CA-3′ and ND5-Phe (V1.04-4) 5′-GTC ATA CTC TAA ATA TAA GCT A-3′ [54] for ND5. Polymerase chain reactions (PCRs) were carried out on a Thermal Cycler TGradient (Biometra GmbH, Göttingen, Germany). The PCR mix of 20 µl contained 1 µl of extracted DNA, 2.5 µl of 10× DreamTaq Green Buffer and 0.4 µl dNTP mixture (10 mM each; both Fermentas GmbH, St. Leon-Rot, Germany), 0.1 µl of each primer (50 pmol/ µl; Biomers GmbH, Ulm, Germany), 0.2 µl DreamTaq Polymerase (5 U/ µl; Fermentas GmbH, St. Leon-Rot, Germany) and 16.2 µl of DNA-free water. Cycling conditions for CO1 started with an initial denaturation for 5 min at 94 °C, followed by 35 cycles of denaturation for 45 at 94°C s, annealing for 45 s at 50°C and extension for 1 min at 72°C. Final elongation was performed for 8 min at 72°C. For ND5 the following PCR program was used: initial denaturation at 94 °C for 5 min, 35 cycles (1 min at 94°, 1 min. at 50°C, 2 min at 70°C), then final elongation for 7 min at 70°C.

PCR products were visualised on 2% agarose gels, dyed with Roti-Safe Gelstain (Carl Roth GmbH, Karlsruhe, Germany) and then purified with the GeneJET PCR Purification Kit (Fermentas GmbH, St. Leon-Rot, Germany). The manufacturer's protocol was slightly modified: only 50 µl elution buffer were used for final elution to ensure a minimum DNA concentration of 100 ng/ µl. Purified PCR products were again checked on agarose gels. Sequencing (both forward and reverse strands) of PCR products was carried out at the laboratory of LGC Genomics (Berlin, Germany) using an Automatic Sequencer 3730 xl (Applied Biosystems, Foster City, USA).

### Sequence alignment and phylogenetic analysis

Forward and reverse sequences were assembled and manually corrected using the software Geneious v4.8.5 [55]. Sequences were aligned and checked for reading frame errors in MEGA5 [56]. Prior to phylogenetic analysis, MrModeltest v2.3 [57] was used to identify the best nucleotide substitution model for each mtDNA fragment (CO1 and ND5) ranked by Akaike Information Criterion (AIC). For each gene, we performed an independent run of 60×10^6^ generations, sampling one tree every 1,000 generations. Based on the selected evolutionary models, we investigated phylogenetic relationships within the species *C. irregularis* (including two outgroups) using Bayesian inference (BI), maximum likelihood (ML) and maximum parsimony (MP). The BI tree was calculated by MrBayes v3.2.1 [58] running 2×10^6^ generations, while reaching an average standard deviation of split frequencies<0.01. The ML method was conducted in RAxML v7.3.0 [59] performing 10,000 thorough bootstrap replicates. MP analysis was carried out in MEGA5 [56] using 5,000 bootstrap replicates. Divergence times were estimated by using strict molecular clocks in BEAST v1.7.1 [60] considering gene-specific mtDNA substitution rates for the two studied loci (1.45% My^−1^ for CO1, and 1.59% My^−1^ for ND5) based on rates determined for other carabid beetle species [51], [61]. Population size through time was applied by a Yule speciation model, and Markov-Chain-Monte-Carlo (MCMC) chain length was set to 200×10^6^, whereas other parameters remained in default settings.

### Intra-specific divergence and genetic diversity

Measures of DNA sequence variation within different hierarchical levels (populations, subclades and clades) and in all sequences together (total) were computed for concatenated sequences and for CO1 and ND5 separately using DnaSP v5.10.01 [62]. Variation measures included the number of polymorphic/segregating sites (S), number of haplotypes (H), haplotype diversity (h) and nucleotide diversity (π). In addition, we performed an analysis of molecular variance (AMOVA) using ARLEQUIN v3.5 [63] to estimate the level of genetic differentiation (Φ_ST_) among populations, subclades and clades. We used the median-joining approach [64] implemented in the NETWORK Software v4.6.1.0 (http://www.fluxus-engineering.com) to illustrate phylogenetic and geographic patterns in haplotype diversity. Intra-specific genetic divergence in CO1 – both within and between major clades – and inter-specific genetic divergence in the studied outgroup species of the subgenus *Platycarabus* were calculated by pairwise comparisons (4,005 intra-specific pairs and 554 inter-specific pairs) using Kimura's two parameter (K-2P) model in MEGA.

## Results

### Current and past predicted distribution

**Figure 2:**
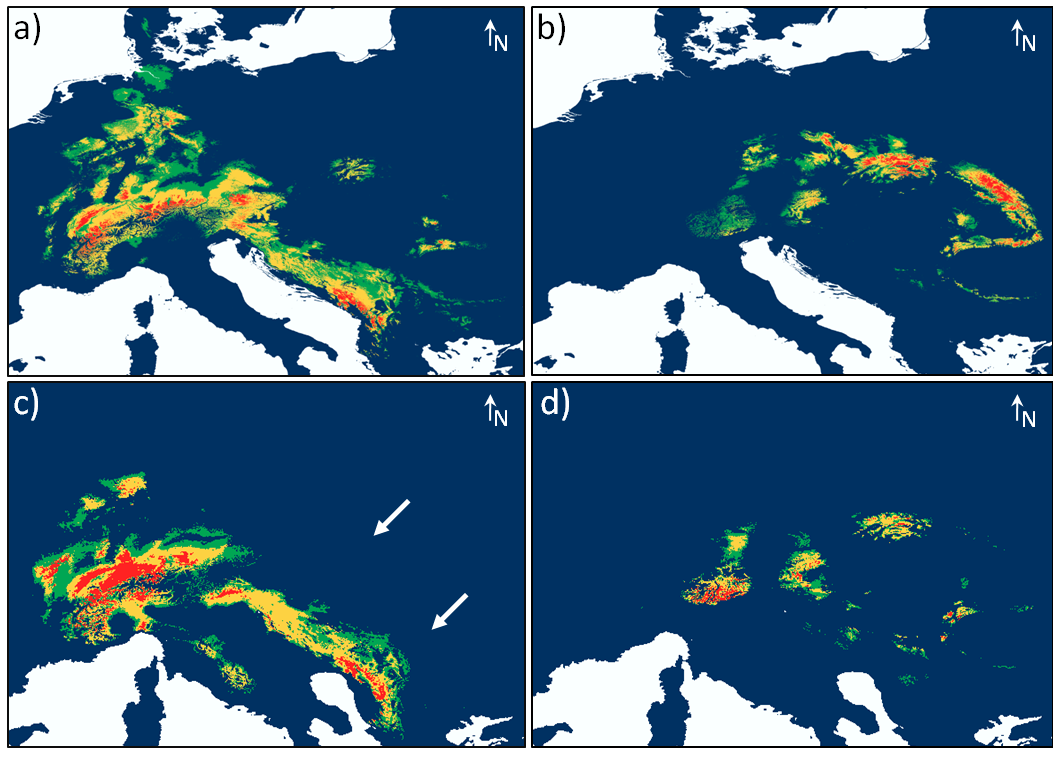
Western European distribution model for (a) current and (c) past climate conditions and Carpathian distribution model for (b) current and (d) past climate conditions. Past distribution is shown for the Last Glacial Maximum (LGM, 21,000 BP). Warmer colors show areas with better predicted conditions (green: p>0.3, yellow: p>0.5, red: p>0.7).

High average training AUC values over all replicate runs for all models – the entire (AUC = 0.92±0.001), the western (AUC = 0.92±0.001) and the Carpathian distribution model (AUC = 0.98±0.002), reflected the high accuracy of our models. Niche overlap was high between the entire distribution model and the western distribution model (*D* = 0.85; *I* = 0.98) and low between the entire and the Carpathian distribution model was low (*D* = 0.39; *I* = 0.71). Lowest niche overlap was found between the western and the Carpathian distribution model (*D* = 0.29; *I* = 0.58). The potential (a) current and (b) past (LGM) distribution of *C. irregularis* computed with the entire distribution model is documented in Fig. S2. Fig. 2 shows maps of the potential (a and b) current and (c and d) past (LGM) distribution of *C. irregularis* calculated with the western and the Carpathian record set, respectively. The SDMs for the western European and the Carpathian populations differed markedly from each other.

The western distribution model for current climate showed the most suitable conditions (p>0.7) in the Jura and Vosges Mountains in eastern France, parts of the Alps (in Switzerland, Germany, Austria and Slovenia), the Southern Carpathians (Transylvanian Alps) and the Apuseni Mountains in Romania as well as in the western Balkan (Dinaric Alps). Moderate suitability (0.5<p<0.7) was found in the lower mountain regions of southern Germany (e.g. Black Forest, Bavarian Forest) and in the low mountain range of northwestern and Central Germany (i.e. Teutoburg Forest, Weser Mountains and Harz) and also in the northern Carpathians (Tatra Mountains). Low suitability (0.3<p<0.5) was attributed to the eastern Carpathians (Romania, Ukraine), the Ardennes (France, Belgium, Luxembourg), large parts of Germany and also up to Denmark. Areas with high suitability rates were always surrounded by areas with lower rates, resulting in a disjunct distribution of suitable habitats. Thus, the model was widely consistent with the documented actual distribution of *C. irregularis*. However, the study species is not known from the regions with low suitability rates, with the exception of the Romanian and Ukrainian Carpathians.

The Carpathian distribution model for current climate conditions (Fig. 2b) displayed the most suitable conditions in parts of the Alps and several high-altitude regions in the Carpathian mountain system.

The past predictive model of *C. irregularis* using the western European data set showed the most suitable conditions (p>0.7) during the LGM along the edges of the large Alpine glacier: in the southwestern Alps, the Vosges and Jura and in the Ligurian part of the Apennines. Well suited climatic conditions were also shown in a large area on the Balkan Peninsula (large parts of the Dinaric Alps) and in a small part of the northwestern German low mountain range. Moderately suitable regions were found in the central Apennines, in the eastern parts of the Alps and in the western Balkan. In the Carpathians, models for LGM conditions showed only small regions with low suitability rates (see arrows in Fig. 2c).

The past distribution model for the Carpathian data set (Fig. 2d) illustrated large areas with highly suitable climate conditions in the southern parts of the Eastern Alps and also high suitability rates for the Tatra Mountains and for some parts of the Southern and the Serbian Carpathians.

### Sequence characteristics

For 97 individuals (including outgroups), the concatenated mtDNA sequence matrix included 1785 bp: partitioned into 786 bp at the CO1 and 999 bp at the ND5 locus. Sequences were submitted to GenBank (http://www.ncbi.nlm.nih.gov/genbank; see GenBank Accession numbers in Table S3). From these mtDNA sequences, we detected 31 haplotypes in *C. irregularis* and 5 in the outgroups (2 *C. depressus*, 3 *C. creutzeri*). In total, 144 sites were variable (67 in CO1, 77 in ND5) and 127 sites were parsimony-informative (CO1: 56, ND5: 71), with no significant difference between the two studied loci (Table S4). Both mitochondrial loci were heavily biased toward high A+T-contents of 71.8% (CO1) and 80.6% (ND5).

### Phylogenetic analyses and divergence time estimation

For both loci, the best evolutionary model identified by the AIC was the general time reversible model with Gamma distributed rate variation across sites and a proportion of invariable sites (GTR+G+I) [65]. Since there has been concern about estimation problems in this model type due to interactions between the proportion of invariable sites and the Gamma distribution [59], we also checked the model without +I and chose this simpler model for the ML analysis in RAxML.

**Figure 3:**
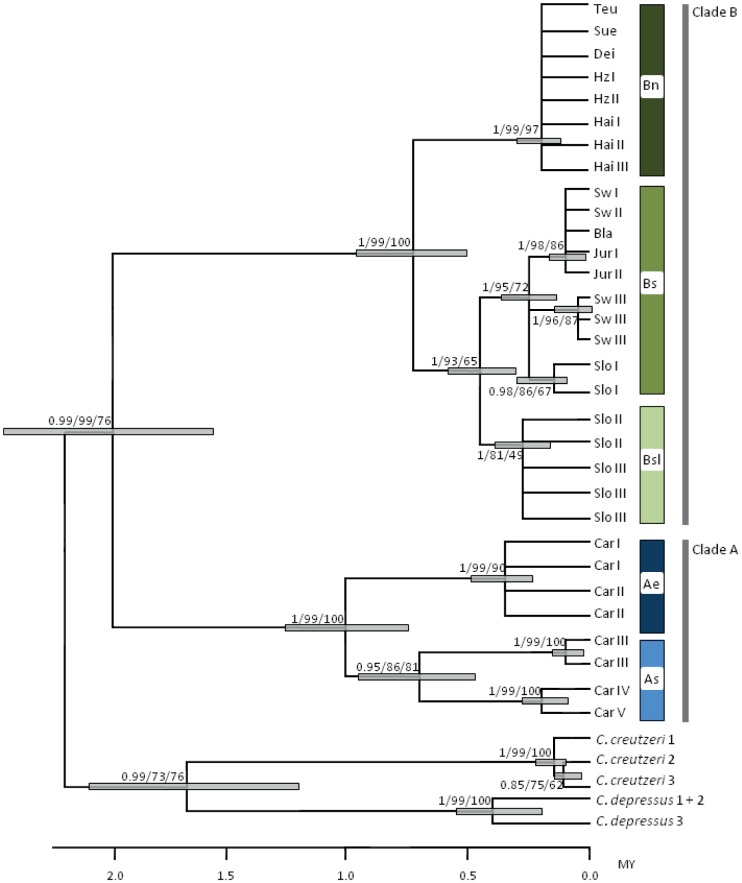
Majority-rule consensus tree for *Carabus irregularis*. The tree shows results for the Bayesian posterior probability (BPP)/the parsimony bootstrap percentages (PB)/the maximum likelihood bootstrap percentages (MLB) for well-supported nodes. Divergence times are displayed with median values and 95% confidence intervals for nodal ages (bars) in million years BP (MY; scale at the bottom). See Table 1 for population abbreviations and Figure 1 for clade abbreviations and colour codes.

BI, MP and ML yielded highly congruent phylogenetic trees with the same major nodes and branching order. The majority-rule consensus tree (Fig. 3) displays the Bayesian posterior probability (BPP), parsimony bootstrap percentages (PB) and maximum likelihood bootstrap percentages (MLB), where BBP ≥ 0.5 and bootstrap values (PB and MLB)≥50% represent well-supported nodes [66]. In addition, nodal ages and 95% confidence intervals are represented for each node.

In the phylogenetic tree, all basal nodes had very high support (BPP≥0.98; PB≥93; MLB≥65) and branches represent two major clades with the following groups of populations: (A) an eastern clade including two Carpathian subclades and (B) a western clade. The eastern clade covers (Ae) an eastern Carpathian subclade (populations Car I and Car II) and (As) a Southern Carpathian subclade (Car III, Car IV and Car V). The western clade comprises (Bn) a northern/central German subclade (Teu, Sue, Dei, Hz I, Hz II, Hai I, Hai II and Hai III), (Bs) a subclade including populations from Southern Germany (Bla, Sw I, Sw II, Sw III), the French Jura mountains (Jur I, Jur II) and Slovenia (Slo I) and (Bsl) a purely Slovenian subclade (Slo II, Slo III; Fig. 3). Assumed divergence times spanning the last 2 million years (Fig. 3) indicated that major clades and subclades within *C. irregularis* were formed long before the last glacial period. The separation of *C. irregularis* from other species of the subgenus *Platycarabus* was found to have taken place earlier than the separation of the species used as outgroups (*C. creutzeri and C. depressus*). Additionally, the split into the two major lineages within *C. irregularis* appears to be older than the split of the two species *C. creutzeri and C. depressus*. Only one of the three subspecies was monophyletic (*C. i. montandoni*), while *C. i. irregularis* and *C. i. bucephalus* are paraphyletic.

### Intra-specific diversity and geographic distribution of genetic diversity

**Figure 4:**
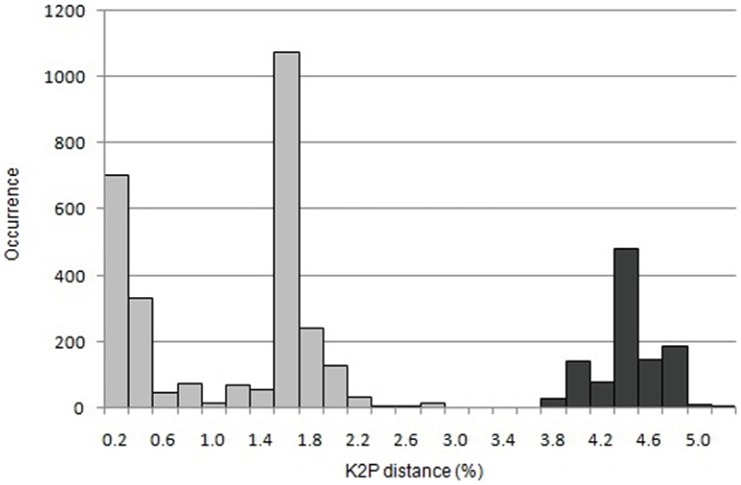
Frequency distribution of intra-specific genetic divergence in CO1 – within (light grey) and between major clades (dark grey). Pairwise distances (4005 intra-specific comparisons within *C. irregularis*) were calculated using Kimura's two parameter (K-2P) model.

The overall haplotype diversity (*h* = 0.882 ± 0.0006) and nucleotide diversity (*π* = 0.017±0.002) were high, with clade A showing higher haplotype and nucleotide diversity (*h* = 0.901; *π* = 0.016) than clade B (*h* = 0.838; *π* = 0.009; Table 2). In general, haplotype and nucleotide diversity tended to be higher in populations from the southern part of the distribution range (Fig. S3). Within clades, subclade As showed higher diversity than Ae and the subclades Bsl and Bs are more diverse than subclade Bn (see Table 3 for a summary of computed diversity measures of different hierarchical levels). The mean intra-specific variation in CO1 (K-2P; *d* = 0.027 ± 0.004) was also very high. The inter-clade K-2P divergence was 0.042 ± 0.004 and the frequency distribution of intra-specific genetic divergence in CO1 within and between major clades showed a clear pattern (Fig. 4). On the population level, K-2P distance values ranged from 0.0 to 0.016 between populations within clade B to 0.022 between populations within clade A. Between populations of clade A and B, genetic distance ranged from 0.040 to 0.044 (Table S5).

**Table 2:** Diversity statistics based on concatinated CO1+ND5 sequences (1785 bp) of *C. irregularis* populations (sampled with more than one specimen) and in subclades and clades (as determined by the phylogenetic tree).

Group	Sample size	Polymorphic sites	Number of haplotypes	Haplotype diversity	Nucleotide diversity
Total	90	144	30	0.882	0.017
Clade A	14	73	8	0.901	0.016
Subclade Ae	7	23	4	0.714	0.005
Car I	2	17	2	1.000	0.010
Car II	5	11	2	0.400	0.002
Subclade As	7	39	4	0.857	0.011
Car III	4	2	2	0.667	0.001
Car V	2	0	1	–	–
Clade B	76	61	22	0.838	0.009
Subclade Bn	38	10	8	0.559	0.001
Sue	6	1	2	0.333	–
Hz I	6	0	1	–	–
Hz II	5	3	2	0.400	0.001
Hai I	6	2	2	0.333	–
Hai II	5	4	4	0.900	0.001
Hai III	4	2	2	0.667	0.001
Subclade Bs	30	17	9	0.667	0.002
Sw I	5	0	1	–	–
Sw II	5	1	2	0.400	0.000
Sw III	4	2	3	0.833	0.001
Bla	6	6	2	0.533	0.002
Ju I	5	1	2	0.400	–
Slo I	4	4	2	0.500	0.001
Subclade Bsl	8	25	7	0.964	0.006
Slo II	4	20	4	1.000	0.007
Slo III	4	11	3	0.833	0.003

**Table 3:** Results of Analysis of Molecular Variance (AMOVA) for different hierarchical levels: subclades, clades and subspecies.

Source of variation	d.f.	Sum of squares	Variation components	Variation (%)	Fixation indices	p value
Among subclades	4	1176.326	18.473	88.100	CT: 0.88100	0.000
Among populations/within subclades	17	151.648	1.944	9.050	SC: 0.65091	0.000
Within populations	68	70.900	1.043	4.860	ST: 0.95144	0.000
Among clades	1	602.756	24.131	72.250	CT: 0.72247	0.000
Among populations/within clades	20	691.222	8.227	24.630	SC: 0.88752	0.000
Within populations	68	70.900	1.043	3.120	ST: 0.96878	0.000
Among subspecies	2	683.794	15.251	63.730	CT: 0.63730	0.000
Among populations/within subspecies	19	610.184	7.637	31.910	SC: 0.87988	0.000
Within populations	68	70.900	1.043	4.360	ST: 0.95643	0.000

**Figure 5:**
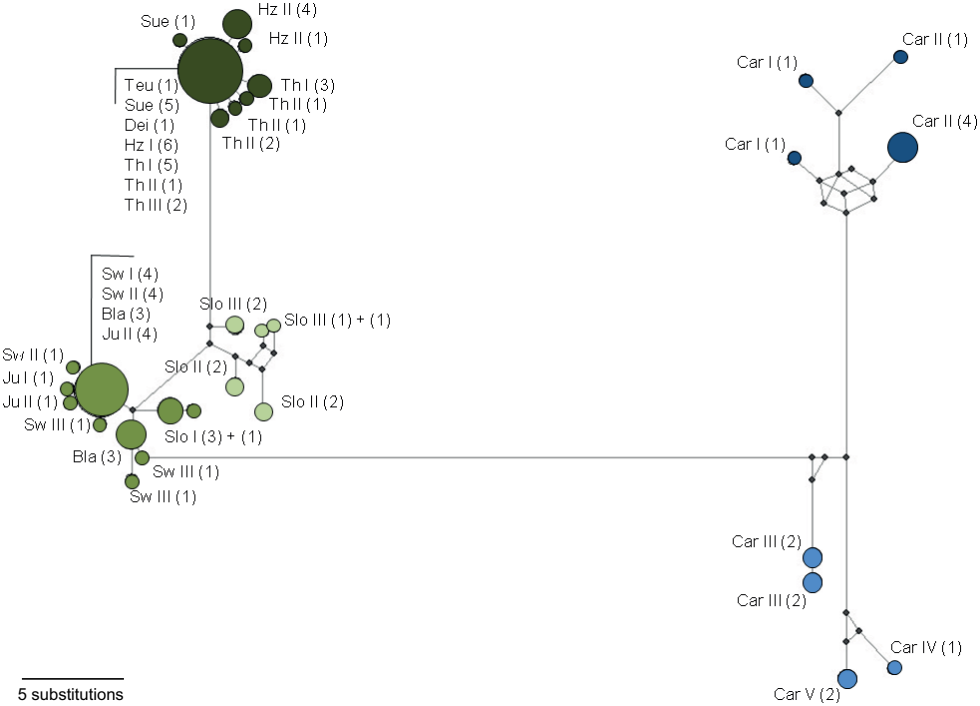
Median-joining network of mtDNA haplotypes based on concatenated CO1+ND5 sequences. Circles represent haplotypes; circle size and numbers in brackets indicate the haplotype frequency within our samples. Small black circles/nodes indicate intermediate haplotypes between observed haplotypes. Haplotype circles are filled corresponding to the colour code for the subclades also used in Figure 1 and 3.

In addition, we detected a significant differentiation structure among all populations (Φ_ST_ = 0.935, p<0.001) and no shared haplotypes between the clades, subclades and subspecies but between populations within clades and subclades. Additionally, AMOVAs for different hierarchical groups (subclades, clades, subspecies) revealed significant genetic variance among these hierarchical groups, among populations and also within populations of the hierarchical groups. Most of the diversity was observed among subclades (88.1%), among clades (72.3%) and among subspecies (63.7%), while low percentage of variance was detected within populations (3.1 to 4.8%; Table 3). Differentiation between the single populations of clade B ranged from lower values in geographically closer populations (Φ_ST_ = 0.10) to higher values in geographically distant populations (Φ_ST_ = 0.99). Populations of clade A also showed very high Φ_ST_ values (>0.9; Table S5), even though the sampled populations were geographically closer than those from clade B. Inter-clade Φ_ST_ values comparing populations of clade A and clade B ranged from 0.92 to 0.99. The haplotype network (Fig. 5) supports these findings and illustrates a similar pattern: high genetic distance between the two geographically distant (∼1,500 km) major clades, but also large genetic distance and a high number of substitutions between the geographically closer (∼300 km) Carphathian subclades (Ae and As). In contrast, populations within subclade Bs showed very low genetic distance, although geographic distance is quite large (between Bla/Sw I-III/Jur I-II and SloI ∼900 km).

## Discussion

### Potential glacial refugia

Our past predictive models showed several independent mountainous regions across Central and southern Europe with highly suitable climate conditions for *C. irregularis* which could have acted as glacial refugia during the LGM. Some of these potential refugia (e.g. in the Dinaric Alps) conformed to classical refugia on the southern European peninsulas [1], [67], [68] and other refugia at the margins of the Alps and in neighbouring mountain ranges (e.g. the Jura, the Black Forest), some of which have quite recently been recognised as refugia [69]–[71]. In addition, our model pointed to refugial areas far north of the southern European peninsulas and the Alps. So-called cryptic refugia have been assumed for a growing group of other species (plants as well as animals) [5], [72].

While the southern European peninsulas are typical glacial refugia of current lowland plant and animal species [2], [73], mountain species potentially persisted in refugia along the border of the Alps when higher altitudes were covered with ice sheets [69], [74]. According to our results, *C. irregularis* seems to have survived in refugia of both low- and high-altitude species. Our models projected relatively large and consistent areas of suitable and moderately suitable conditions during the Last Glacial Maximum (comprising the maximum extension of the continental glaciers), suggesting that *C. irregularis* was relatively widespread during glaciations. In general, numerous mountain species (even though mostly defined as species with their main distribution above the timber line) are assumed to have survived in several spatially restricted and isolated refugia rather than in single extensive and consistent refuge areas ([75] and references therein; [76]). Consequently, *C. irregularis*, a cold-adapted montane to subalpine species, can be expected to have survived the last ice age in multiple spatially restricted refugia within the projected potential distribution area in the Alps and some neighbouring mountain ranges. It appears less important whether there is a significant difference between the historical and the recent distribution range. The fact that the study species tended to have cryptic refugia north of the Alps seems to be of greater importance. While cold-tolerant species, e.g. some mammalian herbivores, have long been believed to have survived in Central European steppe tundra surrounding the glacial ice-sheets, cryptic refugia were surprisingly also found for more temperate animal and tree species [72], [77]. Topographically sheltered sites are assumed to have provided suitable climate conditions and stable microclimates for species with differing habitat requirements [3], [72].

Species distribution modelling is a relatively new but already frequently used method for inferring species potential distribution ranges from occurrence data. The method has been criticised for including abiotic factors only and not taking biotic parameters such as competition and dispersal into account, which might explain discrepancies between the documented range and the projected species range [15], [16]. Nonetheless, the high model quality of our simulations (AUC = 0.92 and 0.98) represented high consistence between actual and predicted occurrence. Another uncertainty, especially of past predictive modelling, is the accuracy of past climate data. We used data processed by the downscaling method, which is currently the best large-scale data available but still requires further validation [18]. Additionally, the modelling approach is based on niche conservatism and does not consider changes in habitat requirements. Nevertheless, past predictive models are useful tools to visualise species distribution patterns and potential glacial refugia. More traditional approaches to identifying refugia are pollen and (sub-)fossil analyses (e.g. [78]). Since no subfossils are known for *Platycarabus* species (cf. [79]), we chose a molecular method (mtDNA analysis) to supplement our findings from modelling and to review assumptions concerning potential glacial refugia.

### Genetic evidence for glacial refugia

Phylogenetic analyses (using BI, MP and ML approaches) revealed two well-supported and spatially congruent major clades: one Central European (B) with three subclades and one Carpathian clade (A) with two subclades. The ages of all major phylogenetic splits predating the last ice age, the absence of shared haplotypes and high genetic intra-specific differentiation between clades as well as subclades implied that the ancestors of phylogenetic (sub)clades in *C. irregularis* survived many glacial-interglacial cycles isolated from each other. The Central European clade consisted of three independent and genetically distinct Central European subclades: one subclade with a more southern distribution (Bs: with populations from southern Germany, France and Slovenia), one Slovenian subclade (Bsl) and another one including haplotypes from the northern part of the species' distribution range (Bn: central to northern Germany).

Thus, the assumption of multiple refugia of *C. irregularis* in Central Europe inferred from past predictive models was supported by our phylogenetic results, with at least two refugia located close to the Alps or on the Balkan Peninsula and one northern refugium, potentially in central Germany. However, phylogenetic analyses indicated the existence of independent Carpathian refugia, which were hardly evident from the western European distribution model for LGM conditions (Fig. 2c, arrows). The past predictive model using the eastern records showed several potential refugia in the Carpathian mountain system (Fig 2d). These potential refugia coincide with the finding of two subclades of clade A one from the eastern (Ae) and one from the southern Carpathians (As), with divergence times dating back more than one million years BP.

As well as refugia in northern Central Europe, Carpathian refugia are not accepted as classical refugial areas and phylogeographic studies including the Carpathian region are still rare. However, since the Carpathians have faced a different glacial history with more local glaciers and larger areas of suitable alpine habitats than the well-studied Alps [9], [80], investigation of the southeastern European mountain range appears likely to be very interesting. Some recent studies (on mountainous plant and animal species) detected low-level differentiation between the Carpathians and the Alps and assumed the Carpathians to have been colonised by adjacent refugia e.g. in the western Alps [8] or the Balkans [71]. In contrast, an increasing number of phylogeographic studies have found very old and divergent phylogenetic lineages, suggesting a major refugium for plants [9], [80] as well as for vertebrates [81]–[84] in the Carpathian region. In congruence with our phylogeographic results for *C. irregularis*, alpine plants, namely *Hypochaeris uniflor* and *Campanula alpina*, showed distinct and strongly differentiated genetic groups within the Carpathians, also pointing to long-term isolation and restricted gene flow between several areas within the mountain system [9], [80], [85]. While Ronikier et al. [80] discovered the strongest differentiation between western and eastern Carpathian samples, results of Mráz et al. (2007) [85] resembled our findings in that they also showed high differentiation between southern and eastern Carpathian populations. Examples from animals also revealed genetically distinct and long-term isolated phylogenetic groups in the Carpathians [81]–[84].

### DNA barcoding and systematic delineation

The phylogenetic results of our study agreed only in part with subspecies systematics: Whereas the Carpathian subspecies *C. i. montandoni* was monophyletic (clade A), the other two subspecies (*C. i. irregularis* and *C. i. bucephalus*) were paraphyletic (clade B) and we suggest to rank the populations belonging to clade B as one subspecies. The high proportion of polymorphic sites within ND5 (7.70%) and also within the CO1 loci (8.52%), which is commonly used as a barcoding marker [19] – also for ground beetles [86], questions the conventional taxonomy. DNA barcoding using the CO1 locus for rapid species delineation [87], implies that inter-clade K-2P distances higher than 2.8 to 3.4% indicate species rather than subspecies or populations. Following these thresholds, the divergence of 0.042 ± 0.004 between clade A (taxonomic subspecies *C. i. montandoni*) and B (*irregularis* s. str.) points to independent species rather than subspecies of the same species. Although the commonly used Kimura's two-parameter substitution model (K-2P) has been repeatedly criticised [88], [89], estimates using other substitution models showed similar results for species delineation [89]. For *C. irregularis*, the calculation of other (simpler as well as more complex) genetic distance metrics shows a consistent pattern with K-2P (*p* distance between clade A and B = 0.042; Tamura-Nei distance between clade A and B = 0.044). However, due to the fact that substitution rates can vary even within the same species [90] it is necessary to exercise caution when defining a species from nucleotide rates alone.

Using morphological and molecular data Casale et al. [91] reconstructed the phylogeny of the subgenus *Platycarabus*. Following their phylogeny *C. irregularis* shows the strongest differentiation from all other species within the subgenus (basal split) and *C. depressus* and *C. creutzeri* are the second major diverged species pair. Surprisingly, the split between the two main lineages of *C. irregularis* we found is older than the divergence between the latter species pair. This underlines both (i) the possible species status of the Carpathian populations and the (ii) need to incorporate a population-based approach to understand the phylogeny of these highly differentiated ground beetles with their ancient lineages, even below or at the species level.

## Conclusion

Past predictive modelling and phylogenetic analyses acted as supplements and imply that *C. irregularis* survived the last glacial periods in long-term isolated classical refugia on the edges of the Alps as well as in other refugia (in Central Europe and some parts of the Carpathian Mountains). Altogether our results indicate that the Carpathian subspecies of *C. irregularis* differs not only in terms of its geographical distribution but also genetically and (due to differing habitat preferences as revealed from the poorly overlapping SDMs) ecologically from rest of the species. Thus we conclude that *C. irregularis* comprises at least two evolutionarily significant units (ESUs, *sensu* Moritz, [92]) indicating that the species has a very interesting history, which should be investigated in more detail by further phylogeographic analyses – also in the framework of the *Platycarabus* group.

## Supporting Information

Figure S1 **Means and 95% confidence intervals of altitudinal distribution of the western and the Carpathian subspecies of** ***Carabus irregularis*** **.**
(EPS)

Figure S2 **Entire distribution model for (a) current and (c) past climate conditions.** Past distribution is shown for the Last Glacial Maximum (LGM, 21,000 BP). Warmer colors show areas with better predicted conditions (green: p>0.3, yellow: p>0.5, red: p>0.7).
(EPS)

Figure S3 **Nucleotide diversity of 19 studied** ***Carabus irregularis*** **populations.** Black circles symbolise values higher than the global mean nucleotide diversity; white circles stand for lower than mean diversity. Circle size is proportional to the difference from the mean diversity. Three populations (Dei, Jur I, Car IV) are omitted due to insufficient sample sizes.
(EPS)

Table S1 **Species records of** ***Carabus irregularis*** **and subspecies affiliation (according to Turin et al. (2003)) used for species distribution modelling.**
(XLSX)

Table S2 **Climate data (monthly temperature and rainfall values) generated according to Hijmans et al. (2005)** [**34**] **to 19 bioclimatic variables.**
(XLSX)

Table S3 **Population and specimen codes and GenBank Accession numbers of the analysed CO1 and ND5 sequences.**
(XLSX)

Table S4 **Summary of statistical analyses on measures of DNA sequence variation within clades, subclades and in all sequences together (total) for CO1 and ND5 separately: N = sample size, S = number of polymorphic/segregating sites, Eta = total number of mutations, k = average pairwise nucleotide difference per sequence, H = number of haplotypes, h = haplotype diversity, π = nucleotide diversity, θG = mutation parameter per sequence).**
(XLSX)

Table S5 **Genetic differentiation (Φ_ST_; in the upper part of the matrix) and intra-specific genetic divergence (K-2P distances; in the lower part of the matrix) of** ***Carabus irregularis*** **.** K-2P distances greater than the threshold value assumed for species delineation by Wiemers and Fiedler [87] are printed in bold.
(XLSX)
